# Ly6c^Lo^ non-classical monocytes promote resolution of rhesus rotavirus-mediated perinatal hepatic inflammation

**DOI:** 10.1038/s41598-020-64158-2

**Published:** 2020-04-28

**Authors:** Anas Alkhani, Claire S. Levy, Margaret Tsui, Katherine A. Rosenberg, Katya Polovina, Aras N. Mattis, Matthias Mack, Steven Van Dyken, Bruce M. Wang, Jacquelyn J. Maher, Amar Nijagal

**Affiliations:** 1 Department of Surgery, University of California, San Francisco, CA USA; 2 Department of Medicine, University of California, San Francisco, CA USA; 3 Department of Pathology, University of California, San Francisco, CA USA; 4 The Liver Center, University of California, San Francisco, CA USA; 5 Regensburg University Medical Center, Regensburg, Germany; 6 Department of Pathology and Immunology, Washington University School of Medicine, Saint Louis, MO USA

**Keywords:** Inflammation, Innate immune cells

## Abstract

Perinatal hepatic inflammation can have devastating consequences. Monocytes play an important role in the initiation and resolution of inflammation, and their diverse functions can be attributed to specific cellular subsets: pro-inflammatory or classical monocytes (Ly6c^Hi^) and pro-reparative or non-classical monocytes (Ly6c^Lo^). We hypothesized that inherent differences in Ly6c^Hi^ classical monocytes and Ly6c^Lo^ non-classical monocytes determine susceptibility to perinatal hepatic inflammation in late gestation fetuses and neonates. We found an anti-inflammatory transcriptional profile expressed by Ly6c^Lo^ non-classical monocytes, and a physiologic abundance of these cells in the late gestation fetal liver. Unlike neonatal pups, late gestation fetuses proved to be resistant to rhesus rotavirus (RRV) mediated liver inflammation. Furthermore, neonatal pups were rendered resistant to RRV-mediated liver injury when Ly6c^Lo^ non-classical monocytes were expanded. Pharmacologic inhibition of Ly6c^Lo^ non-classical monocytes in this setting restored susceptibility to RRV-mediated disease. These data demonstrate that Ly6c^Lo^ monocytes promote resolution of perinatal liver inflammation in the late gestation fetus, where there is a physiologic expansion of non-classical monocytes, and in the neonatal liver upon experimental expansion of these cells. Therapeutic strategies directed towards enhancing Ly6c^Lo^ non-classical monocyte function may mitigate the detrimental effects of perinatal liver inflammation.

## Introduction

Liver inflammation can have severe, life-threatening consequences, particularly in neonates. The progressive nature of perinatal inflammation is at odds with the known anti-inflammatory and tolerogenic immune environment that exists in the late gestation fetal environment^1–3^. These observations suggest that the aggressive nature of liver inflammation that occurs in neonates results from impairment of the physiologic reparative mechanisms that exist during perinatal development.

Monocytes are innate immune cells that circulate through the bloodstream and migrate to inflamed and/or injured tissues. Among the initial wave of leukocytes recruited to the hepatic parenchyma during liver inflammation^4^, monocytes play an important role during the initiation and resolution of the inflammatory response in injured tissues^5^. Monocytes can be separated into two main subsets based on cell surface markers. In mice, classical monocytes express high levels of Ly6c, whereas non-classical monocytes express low levels of Ly6c^5^. In addition to Ly6c, the differential expression of C-C motif chemokine receptor 2 (Ccr2) and C-X3-C motif Chemokine Receptor 1 (Cx3cr1) separates these two monocyte subsets: Ly6c^Hi^ classical monocytes are Ly6c^Hi^Ccr2^Hi^Cx3cr1^int^ and Ly6c^Lo^ non-classical monocytes are Ly6c^Lo^Ccr2^Lo^Cx3cr1^Hi^^5^. Current studies support the idea that circulating Ly6c^Hi^ classical monocytes are recruited to sites of inflammation and injury, where they perform pro-inflammatory functions^4,6,7^. It is also believed that Ly6c^Hi^ classical monocytes transition into Ly6c^Lo^ non-classical monocytes, which then promote anti-inflammatory and pro-reparative functions during inflammation^5^. In the physiologic state, Ly6c^Lo^ non-classical monocytes also act as vascular scavengers, surveilling the endothelium and eliminating luminal microparticles^8,9^. The functions of monocytes during perinatal liver development and their contributions to the pathogenesis and resolution of perinatal hepatic inflammation are unknown.

The purpose of this study was to define the roles of Ly6c^Hi^ classical monocytes and Ly6c^Lo^ non-classical monocytes during perinatal hepatic inflammation. Based on our understanding of monocyte subsets in adult tissues, we hypothesized that inherent differences in Ly6c^Hi^ classical monocyte and Ly6c^Lo^ non-classical monocyte function and their relative abundance in the liver determine susceptibility to hepatic inflammation in late gestation fetuses and neonates. To test this hypothesis, we performed single cell RNA sequencing to define the transcriptional profile of these cells, and quantified Ly6c^Hi^ classical monocytes and Ly6c^Lo^ non-classical monocytes during perinatal liver development. Using a mouse model of perinatal hepatic inflammation, we determined that the abundance of Ly6c^Lo^ monocytes in the liver inversely correlates with disease susceptibility. Moreover, we demonstrated that expansion of Ly6c^Lo^ non-classical monocytes in perinatal mice renders animals resistant to an infectious insult. With this approach, we have defined an important role for Ly6c^Lo^ non-classical monocytes in the resolution of perinatal liver inflammation.

## Results

### Single-cell transcriptomic analysis of murine perinatal hepatic monocytes identifies a pro-inflammatory signature among Ly6c^Hi^ classical monocytes and an anti-inflammatory signature among Ly6c^Lo^ non-classical monocytes

**Figure 1:**
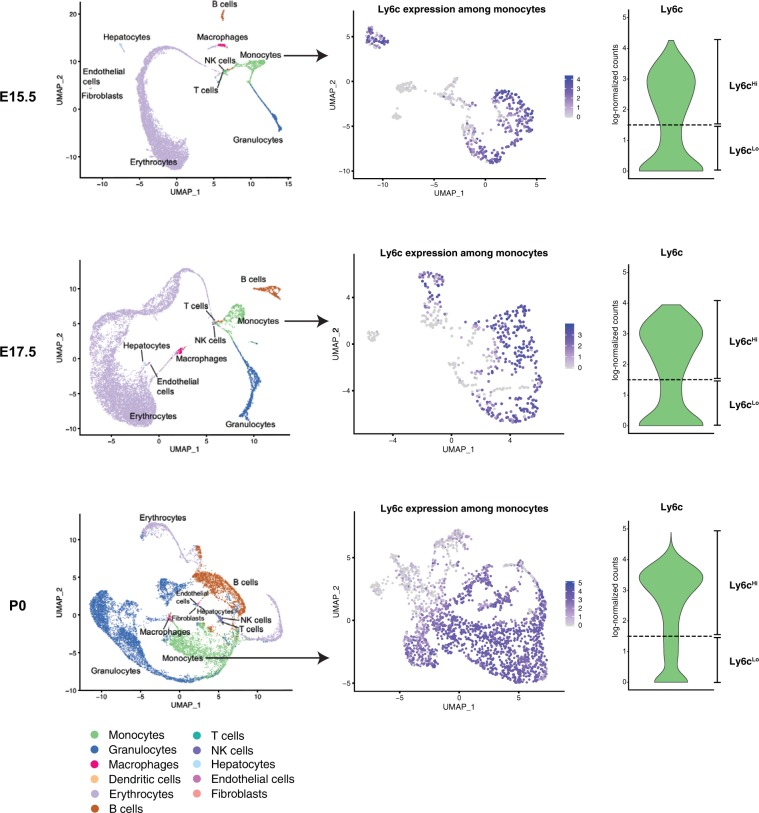
scRNAseq of murine perinatal immune cells and separation of classical and non-classical monocytes based on Ly6c expression. Single cell RNA sequence analysis was performed of the perinatal liver at E15.5, E17.5, and P0. Left panel: Using the SingleR algorithm, perinatal hepatic immune and non-immune populations were defined. Middle panel: Monocytes were identified and further sub-clustered. Right panel: Monocytes with *Ly6c2* expression >1.5 (log-normalized counts) were classified as Ly6c^Hi^ classical monocytes; those with *Ly6c2* expression less than 1.5 (log-normalized counts) were classified as Ly6c^Lo^ non-classical monocytes. Dashed line denotes gene expression at 1.5x log-normalized counts.

Our current understanding of murine monocyte subsets supports the idea that Ly6c^Hi^ classical monocytes are pro-inflammatory and Ly6c^Lo^ non-classical monocytes are anti-inflammatory/pro-reparative^5^. Studies elucidating the function of monocyte subsets have predominantly focused on adult mice, and little is known about their function during perinatal development. We used single cell RNA sequencing of fetal and neonatal livers to compare the transcriptional profile of hepatic Ly6c^Hi^ classical monocytes and Ly6c^Lo^ non-classical monocytes during perinatal development. Using the SingleR algorithm^10^, we categorized perinatal hepatic immune populations **(**Fig. 1**)**. At E15.5 and E17.5, the majority of cells were identified as erythrocytes and a fraction of cells were identified as monocytes. The prevalence of immune populations, including monocytes, increased in the P0 liver.

**Figure 2:**
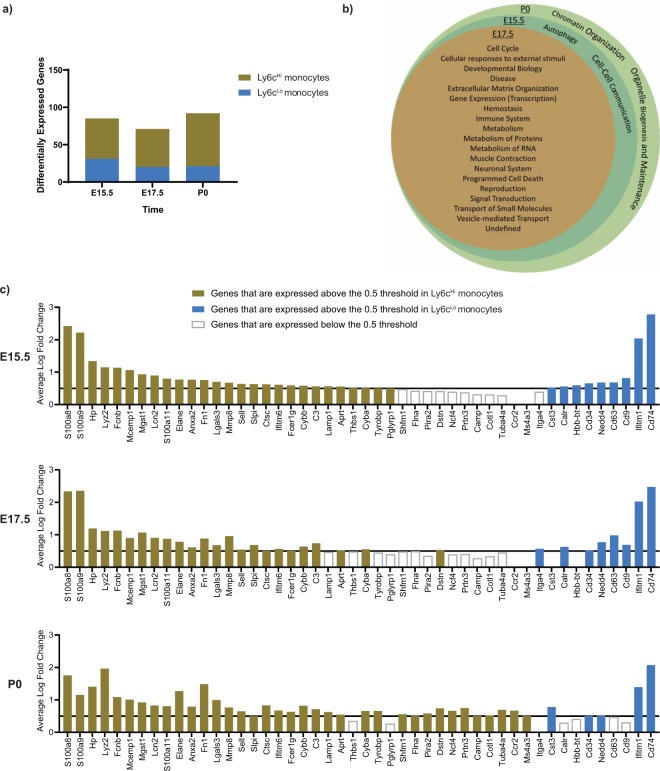
Functional annotation of scRNAseq analysis of murine perinatal hepatic monocytes demonstrates differential expression of several genes in immune system pathways among Ly6c^Hi^ and Ly6c^Lo^ monocytes. (**a**) Number of differentially expressed genes between Ly6c^Hi^ classical monocytes and Ly6c^Lo^ non-classical monocytes from each timepoint (E15.5, E17.5, and P0) and (**b**) Venn diagram of functional pathway annotations. **(c**) Expression of genes of interest in immune-related functional pathways defined in (**a**).

We analyzed monocytes that were identified by SingleR, and separated them by Ly6c expression into Ly6c^Hi^ classical monocytes and Ly6c^Lo^ non-classical monocytes **(**Fig. 1**)**. Of note, Ly6c expression was highest among monocytes and granulocytes with low levels of expression among B- and natural killer cells **(**Supplementary Fig. 1**)**. The separation of classical and non-classical monocytes using Ly6c expression was corroborated by higher levels of Ccr2 and Cd62l among Ly6c^Hi^ monocytes and higher levels of Cx3cr1 among Ly6c^Lo^ monocytes^5^ **(**Supplementary Fig. 2**)**. Using an unbiased approach, we identified numerous genes that were differentially expressed between Ly6c^Hi^ classical monocytes and Ly6c^Lo^ non-classical monocytes (E15.5, 85 genes; E17.5, 71 genes; P0, 92 genes, Fig. 2a**)**. We categorized these differentially expressed genes by identifying the cellular pathways in which they are involved using the REACTOME database^11^ **(**Fig. 2b**)**. Ly6c^Hi^ classical monocytes and Ly6c^Lo^ non-classical monocytes had differential expression of genes involved with immune function at all three time points, along with basic cellular functions including metabolism and signal transduction **(**Fig. 2b**)**. Ly6c^Lo^ monocytes had elevated expression of interferon-induced transmembrane protein 1 (*Ifitm1*), which is involved with the antiviral response to limit viral entry into the cytosol, and *Cd74*, a receptor for Mif that is responsible for initiating cell survival pathways and cellular proliferation **(**Fig. 2c**)**. These transcripts were expressed at high levels in Ly6c^Lo^ non-classical monocytes at all three time points. Several pro-inflammatory genes were expressed at a higher level in Ly6c^Hi^ classical monocytes than in Ly6c^Lo^ non-classical monocytes, including members of the s100 calcium-binding protein family A8, A9, and A11, and ficolin-beta (*Fcnb*). We also examined known pro-inflammatory and pro-reparative mediators, and found elevated expression of anti-inflammatory and pro-reparative genes (*Il4ra* and *Tgfb1*) in Ly6c^Lo^ monocytes, and elevated levels of pro-inflammatory genes (*Mmp8, S100a1, Irf5, Naip2, Casp1, Card9, Ifngr1, Ifngr2, Ifi204, Ifitm2, Ifitm3*, and *Ifitm6*) in Ly6c^Hi^ monocytes **(**Supplementary Fig. 3**)**. The lower levels of pro-inflammatory mediators and higher expression of anti-inflammatory/reparative transcripts in Ly6c^Lo^ monocytes support the hypothesis that these cells may promote resolution of perinatal liver inflammation.

### Ly6c^Lo^ non-classical monocytes outnumber Ly6c^Hi^ classical monocytes in the late gestation fetus

**Figure 3:**
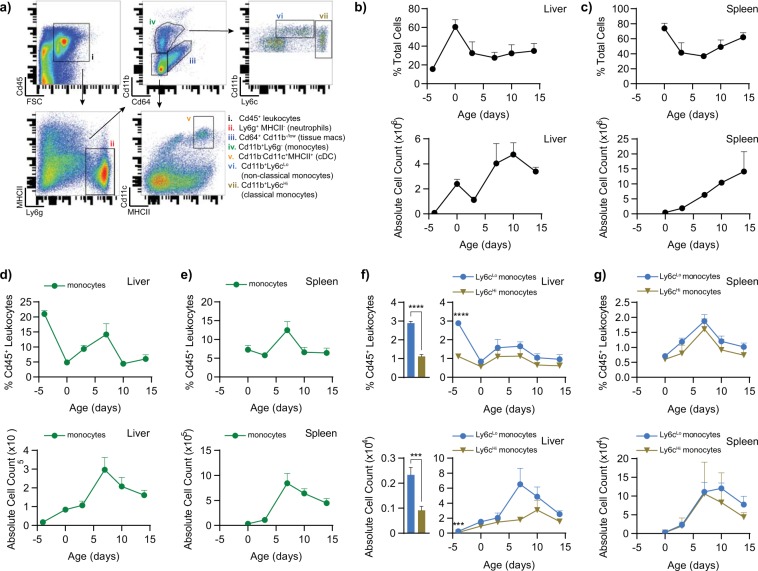
Physiological abundance of Ly6c^Lo^ non-classical monocytes compared to Ly6c^Hi^ classical monocytes in the late gestation fetal liver. (**a**) Ly6c^Lo^ monocytes, Ly6c^Hi^ monocytes, and other leukocyte populations were identified by flow cytometry. (**b,c**) The overall proportion of Cd45^+^ leukocytes is relatively low in the late gestational fetal liver (E17.5, n = 11), then increases by day of birth (P0, n = 9), and plateaus (P3, n = 6; P7, n = 9; P10, n = 6; P14, n = 7), whereas the proportion in the spleen stays relatively stable (P0, n = 6; P3, n = 6; P7, n = 9; P10, n = 6; P14, n = 7. (**d,e**) Cd11b^+^ monocytes were identified and are at their highest abundance of the Cd45^+^ leukocyte compartment in the late gestational liver (E17.5, n = 11). They then decrease at birth (P0, n = 6), rise again to a level that is between the two earlier time points (P3, n = 6; P7, n = 9), and then decrease to day of birth levels (P10, n = 6; P14, n = 4). In the spleen, Ly6c^Lo^ non-classical monocytes remained low at birth and peaked at P7. (f-g) There is a physiological abundance of Ly6c^Lo^ non-classical monocytes compared to Ly6c^Hi^ classical monocytes at E17.5 in the liver that is not observed in the spleen (p-value_%_: <0.0001 (****), p-value_#_: 0.0007 (***)). Data represent mean +/− SEM.

We then quantified the proportion of Ly6c^Hi^ classical monocytes and Ly6c^Lo^ non-classical monocytes in the liver to determine the relative abundance of both populations during perinatal development. To define the proportion of monocyte subsets, we first characterized leukocytes, including monocytes, tissue macrophages, conventional dendritic cells, and neutrophils residing in the livers of late gestation fetuses and neonatal pups **(**Fig. 3a, Supplementary Fig. 4**)**. As a reference lymphoid organ, we also examined the leukocyte composition of the spleen during these time points (the spleen was not visible in the late gestation, E17.5 fetus). Cd45^+^ leukocytes comprised 15.8% of the total cells in the E17.5 fetal liver with the proportion of leukocytes increasing to 60.7% of total cells at the time of birth, likely resulting from the rapid expansion of hematopoietic progenitor cells that occurs in the late gestation fetal liver **(**Fig. 3b**)**^12^. The absolute number of leukocytes also fluctuated during this period, peaking at day 10. Leukocyte percentages in the spleen ranged between 73.8% and 61.9%, with a nadir 7 days after birth (36.8%) **(**Fig. 3c**)**. In concordance with its development as a lymphoid organ during postnatal life, the cellularity of the spleen increased through all the time points tested. Cd45^+^Cd11b^+^Ly6g^-^Cd64^-^ monocytes comprised ~21.0% of the total leukocyte pool in the late gestation fetal liver **(**Fig. 3d**)**. Total monocyte percentages were high in the late gestation fetus, decreased at birth (4.9%), re-expanded, albeit to lower levels than at E17.5 (14.2%), and decreased again after day 7 to 6.0%. The absolute number of monocytes increased in the liver with a peak at day 7, likely reflecting the expansion of leukocytes seen in the postnatal liver **(**Fig. 3b**)**. We noticed a similar trend in the spleen, as monocyte percentages remained low at birth (7.3%) and at P3 (5.8%), before spiking at day 7 (12.5%) and plateauing at day 14 (6.4%) **(**Fig. 3e**)**. The absolute number of monocytes in the spleen mirrored the proportion of monocytes, also peaking at day 7.

Given the importance of monocyte subsets in the initiation and resolution of inflammation, and their abundance in the late gestation fetus, we quantified the proportions of Ly6c^Lo^ non-classical monocytes and Ly6c^Hi^ classical monocytes during late gestation fetal and neonatal development. We observed that Ly6c^Lo^ non-classical monocytes are the predominant monocyte population in late gestation fetal liver **(**Fig. 3f**)**. Whereas Ly6c^Hi^ classical monocytes account for 1.1% (±0.10%) of total leukocytes at E17.5, Ly6c^Lo^ non-classical monocytes comprise 2.9% (±0.10%) of leukocytes. This difference in the proportions of monocyte subsets was reflected by the absolute numbers of these populations, demonstrated by a significantly higher number of Ly6c^Lo^ monocytes than Ly6c^Hi^ monocytes in the late gestation fetal liver. The proportions of each of these cell types equalized at the time of delivery in the liver **(**Fig. 3f**)**, approaching the eventual 1:1 ratio of Ly6c^Lo^:Ly6c^Hi^ monocytes that exists in circulation **(**Fig. 3g**)**^5^.

### The physiological abundance of Ly6c^Lo^ non-classical monocytes correlates with resistance to periportal inflammation

**Figure 4:**
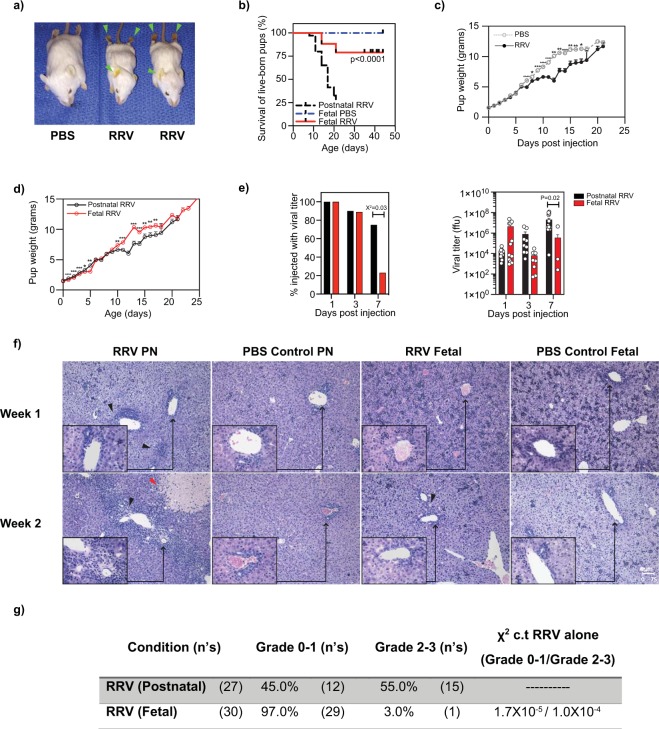
The physiological abundance of Ly6c^Lo^ non-classical monocytes in the late gestation fetus inversely correlates with susceptibility to periportal inflammation. (**a**) Images of P14 Balb/c pups demonstrate the phenotypic changes (jaundice, growth restriction, and weight loss) associated with postnatal RRV-infection compared to PBS controls. (**b**) Survival of live-born pups after fetal RRV infection was significantly higher than for neonates infected with RRV (postnatal RRV: n = 15/73, 20% survival, Chi-square = 1.1 × 10^−11^ c.t. PBS controls; fetal RRV: n = 38/43, 88% survival, Chi-square = 1.0 × 10^−22^ c.t. postnatal RRV; Kaplan-meier survival curve, fetal RRV c.t. postnatal RRV, p-value <0.0001). (**c**) RRV-infected pups experienced significant weight loss (n = 73) compared to PBS controls (n = 27). (**d**) Unlike animals that undergo postnatal RRV infection (n = 73), fetuses infected with RRV do not experience neonatal growth restriction. Data represent mean +/− SEM. (p-value: <0.05 (*), <0.01 (**), <0.001 (***), <0.0001 (****)). (**e**) Viral titers from infected livers were calculated to determine the proportion of livers that had infectious viral particles. Quantification of viral titers was also determined in those livers that had infectious particles. Data represent at least 2 independent experiments and at least 9 animals per group. Error bars represent the mean +/- SEM. (**f**) Hematoxylin & Eosin (H&E) stained sections demonstrate higher grade of periportal inflammation and necrotic foci formation after RRV-mediated liver injury in postnatal pups one week (n = 6) and two weeks (n = 4) after infection compared to fetal RRV infection, one week (n = 6) and two weeks after infection (n = 2). Fetal RRV samples demonstrate a similar degree of extramedullary hematopoiesis in the early neonatal stage when compared to PBS controls. Black arrows point to periportal inflammation in liver samples after RRV-mediated liver injury. Red arrow points to necrotic foci in liver samples after RRV-mediated liver injury. (**g**) Grading of periportal inflammation in liver tissue samples after postnatal (n = 27) and fetal (n = 30) RRV infection.

The anti-inflammatory transcriptional profile of perinatal Ly6c^Lo^ non-classical monocytes and their relative abundance at E17.5 led us to question whether Ly6c^Lo^ non-classical monocytes confer resistance to perinatal inflammation in the late gestation fetus. To study the role of Ly6c^Hi^ classical monocytes and Ly6c^Lo^ non-classical monocytes during perinatal hepatic inflammation, we used rhesus rotavirus (RRV) infection to induce perinatal liver injury and to determine whether physiological differences in the abundance of Ly6c^Lo^ non-classical monocytes and Ly6c^Hi^ classical monocytes affects susceptibility. The RRV model of perinatal liver inflammation has been used to study the mechanism(s) of immune-mediated periportal inflammation and bile duct obstruction in neonatal animals^13^. Similar to previously published results, intraperitoneal injection of 1.5 × 10^6^ focus-forming units (ffu) RRV during the first 24 hours of life resulted in the development of jaundice **(**Fig. 4a**)** and death in the majority of animals (79% mortality, median survival 17 days, Fig. 4b**)**^13,14^. Neonatal pups experienced progressive weight loss 7 days after RRV infection compared to PBS controls **(**Fig. 4c**)**. Infected animals developed periportal inflammation 7 days after infection, with progression to tissue necrosis until the majority of animals succumbed to their illness (Fig. 4b,f). This infectious model of periportal inflammation resulted in inflammation only in the liver, without affecting other organs **(**Supplementary Fig. 5**)**. Of note, there was no inflammation or mucosal sloughing of the small and large intestine observed, despite the known effects of rotavirus on the gastrointestinal system **(**Supplementary Fig. 5**)**^15^.

To determine whether the relative abundance of Ly6c^Lo^ non-classical monocytes we identified in the late gestation fetus is associated with resistance to RRV-mediated liver inflammation, we adapted the neonatal model of RRV infection to the fetal environment using fetal surgical techniques that we have previously described^16,17^. E14.5 fetuses were infected with RRV and subsequently tracked for survival and development of liver disease. Over 75% of live-born mice infected with RRV in utero (79.1%, n = 38/43) lived beyond 21 days, which was significantly higher than the proportion surviving after postnatal RRV infection (p < 0.0001, Fig. 4b). In contrast to the weight loss we observed in RRV-infected neonatal pups **(**Fig. 4c**)**, fetuses injected with RRV did not develop neonatal growth restriction **(**Fig. 4d**)**, corroborating our findings that fetuses exposed to RRV are resilient to RRV-mediated injury. We confirmed that the improved survival of prenatally-infected mice was not due to a failure of RRV infection: indeed, a similar proportion of animals that underwent fetal and postnatal RRV infection had a positive viral titer 3 days after infection, and viral titers in the liver were initially similar after fetal RRV infection **(**Fig. 4e**)**. Histological analysis of livers 2 weeks after RRV infection showed minimal periportal inflammation after fetal infection compared to postnatal infection **(**Fig. 4f**)**. We compared the grade of inflammation after postnatal and fetal RRV infection in a blinded fashion and found fewer mice with grade 2–3 inflammation after fetal RRV infection **(**Fig. 4g**)**. A similar degree of extramedullary hematopoiesis was present in fetal livers 1 week after RRV infection and in fetal PBS control livers **(**Fig. 4f**)**. Additionally, we found fewer necrotic foci after fetal RRV infection compared to postnatal infection (data not shown). These data demonstrate that fetuses are resistant to RRV-mediated infection despite effective delivery of RRV and establishment of infection, supporting our hypothesis that physiologic differences in monocyte composition may account for resilience in the late gestation fetus.

### T- and B-lymphocytes are not required for RRV-mediated inflammation, and may play a protective role during the later stages of RRV-mediated inflammation

**Figure 5:**
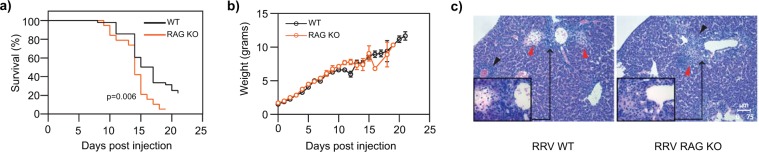
Adaptive immune cells play a protective role during the later stages of RRV-mediated inflammation. (**a**) Kaplan-Meier survival after RRV infection in WT (n = 40) and RAG KO (n = 19) animals. RAG KO animals have similar rates of survival compared to WT during the first 2 weeks of infection. There is an increase in mortality in RAG KO animals that is apparent during the latter phase of RRV infection (p-value 0.006). (**b**) Weight over time of WT pups injected with RRV (n = 73) compared to RAG KO pups injected with RRV (n = 19). Data represent the mean +/−SEM. (**c**) Hematoxylin and eosin (H&E) stained sections demonstrate histological changes within the liver tissue of postnatal pups two weeks after injection with RRV, (left to right): WT (n = 4), RAG KO (n = 5). Black arrows point to periportal inflammation in liver samples after RRV-mediated liver injury. Red arrow points to necrotic foci in liver samples after RRV-mediated liver injury.

Our data support the role of myeloid populations, and specifically monocytes, in the pathogenesis of RRV-mediated inflammation. In addition to the myeloid compartment, other immune mediators, including type 2 immune responses and the adaptive immune system, have been implicated in the pathogenesis of RRV-mediated inflammation^14,18–21^. Type 2 immunity was not required for establishing disease after RRV infection, as mice deficient in thymic stromal lymphopoietin receptor, IL-33 receptor, and IL-25 had similar survival, weight loss, and inflammation when compared to wildtype controls **(**Supplementary Fig. 6**)**. To determine whether T- and B-cells contribute to the pathogenesis of RRV-mediated perinatal liver inflammation, we infected neonatal RAG KO (deficient in T- and B-lymphocytes) pups with RRV. We found that there was no difference in survival during the first 2 weeks after infection compared to immunocompetent wildtype animals **(**Fig. 5a**)**. RAG KO animals did, however, have a survival disadvantage 14 days after RRV infection. This pattern of mortality is in stark contrast to that of RRV-infected fetuses, for which there is a survival advantage in the early stages of infection, thus supporting the idea that monocytes may impact disease susceptibility during the early phase of RRV-mediated inflammation. Similar to wildtype mice, RAG KO animals experienced weight loss and periportal inflammation **(**Fig. 5b,c**)**. Collectively, these data support a limited role of the adaptive immune system and type 2 immunity in the pathogenesis of RRV-mediated inflammation.

### Experimental ablation of Ly6c^Hi^ classical monocytes and neutrophils results in the expansion of Ly6c^Lo^ non-classical monocytes during RRV-mediated periportal inflammation and confers protection from disease

**Figure 6:**
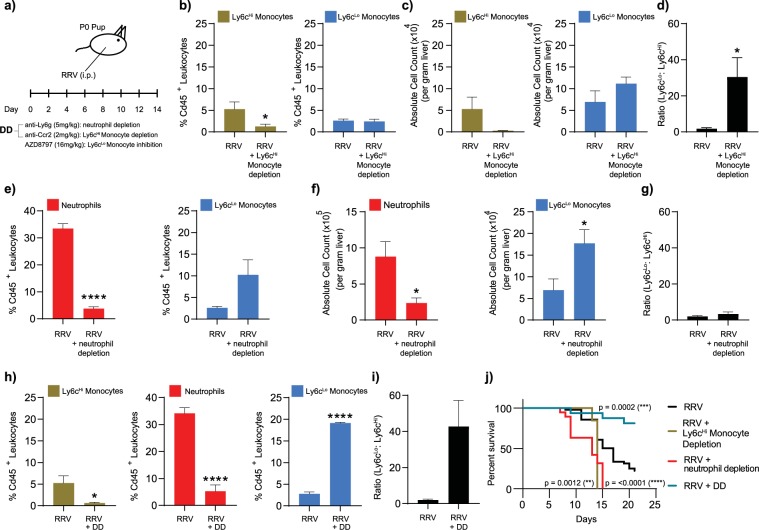
Ly6c^Lo^ non-classical monocytes promote resolution of RRV-mediated periportal inflammation. (**a**) Dosage schedule illustrating regimen used to deplete Ly6c^Hi^ classical monocytes (anti-Ccr2) and neutrophils (anti-Ly6g), and to inhibit Ly6c^Lo^ non-classical monocytes (Cx3cr1 small molecular inhibitor, AZD8797) after inducing RRV-mediated liver injury in neonatal pups. (**b–d**) Depletion via anti-Ccr2 led to a 76% reduction of Ly6c^Hi^ classical monocytes in the Cd45^+^ leukocyte compartment, which did not affect the proportion of Ly6c^Lo^ non-classical monocytes (RRV n = 11, RRV_anti-Ccr2_ n = 10) and a 95% reduction of the absolute number of Ly6c^Hi^ classical monocytes expected in a P7 liver. This led to a statistically significant increase in the ratio of Ly6c^Lo^: Ly6c^Hi^ monocytes (p-value 0.026 (*)). (**e-g**) Depletion of neutrophils alone (p-value_E_: <0.0001 (****), p-value_F_: 0.048 (*)) led to an expansion of Ly6c^Lo^ non-classical monocytes, (p-value_F_: 0.04 (*)), but did not change the ratio of Ly6c^Lo^: Ly6c^Hi^ monocytes ((**d**) RRV n = 14, RRV_anti-Ly6g_ n = 7, (**e**) RRV n = 4, RRV_anti-Ly6g_ n = 4). (**h–j**) Depleting both Ly6c^Hi^ classical monocytes (p-value: 0.0207 (*)) and neutrophils (p-value: <0.0001 (****)) (Double Depletion, DD) led to further expansion of Ly6c^Lo^ non-classical monocytes (p-value: <0.0001 (****)) (n = 4) and a significantly higher survival rate when compared to RRV alone (p-value 0.0002) or RRV plus single depletion (RRV n = 49, RRV _Ly6c_^Hi^_classical monocytes depletion_ n = 13, RRV_neutrophil depletion_ n = 19, RRV_DD_ n = 16). Data represent mean +/− SEM. (p-value: <0.05 (*), <0.0001 (****)).

Having demonstrated a correlation between the physiologic abundance of Ly6c^Lo^ non-classical monocytes in the late gestation fetus and resistance to periportal hepatic inflammation, we hypothesized that if Ly6c^Lo^ monocyte numbers in the neonatal liver could be increased experimentally to prenatal levels, then postnatal animals would be rendered similarly resistant to perinatal liver inflammation. Using antibodies that target receptors expressed on Ly6c^Hi^ monocytes and neutrophils, we depleted these two pro-inflammatory populations individually and quantified the effects on Ly6c^Lo^ non-classical monocytes **(**Fig. 6a,b**)**. In addition to expressing Ly6c, Ly6c^Hi^ monocytes also express high levels of Ccr2. This cell surface molecule can be used as a target for antibody-mediated depletion of Ly6c^Hi^ classical monocytes^22^ **(**Supplementary Fig. 7**)**. Administration of anti-Ccr2 (clone MC21) in the setting of RRV infection led to the elimination of >60% of Ly6c^Hi^ classical monocytes compared to RRV-infected livers without Ly6c^Hi^ classical monocyte depletion **(**Fig. 6b,c**)**. We examined the impact of Ly6c^Hi^ classical monocyte depletion on hepatic Ly6c^Lo^ non-classical monocytes, and observed that even though the proportion of Ly6c^Lo^ monocytes did not change, the Ly6c^Lo^: Ly6c^Hi^ monocyte ratio increased **(**Fig. 6d**)**.

Anti-Ly6g (clone 1A8, BioX-cell) was used to deplete neutrophils, and resulted in ~90% reduction of neutrophils during RRV infection **(**Fig. 6e, Supplementary Fig. 7**)**. When neutrophils were depleted, we observed an increase in the proportion of Ly6c^Lo^ non-classical monocytes, although it was not statistically significant **(**Fig. 6e**)**. We did, however, observe a significant increase in the absolute number of Ly6c^Lo^ non-classical monocytes in RRV-infected livers when neutrophils were depleted **(**Fig. 6f**)**. This expansion did not result in a change in the Ly6c^Lo^: Ly6c^Hi^ monocyte ratio (Fig. 6g**)**. We predicted that if both neutrophils and Ly6c^Hi^ classical monocytes were depleted simultaneously, than we would observe an increase in both the absolute number of Ly6c^Lo^ non-classical monocytes and the ratio of Ly6c^Lo^: Ly6c^Hi^ monocytes. Indeed, when Ly6c^Hi^ classical monocyte - and neutrophil-depleting antibodies were administered simultaneously (double depletion, DD), there was a significantly higher proportion of Ly6c^Lo^ non-classical monocytes in RRV-infected livers **(**Fig. 6h**)**. Furthermore, the ratio of Ly6c^Lo^: Ly6c^Hi^ monocytes was higher after the simultaneous depletion of neutrophils and Ly6c^Hi^ classical monocytes (RRV alone 1.6; RRV with DD 42.8; Fig. 6i), thereby resembling the late gestation fetal liver where there is an abundance of Ly6c^Lo^ non-classical monocytes. Of note, simultaneous depletion of neutrophils and Ly6c^Hi^ classical monocytes had no statistically significant effect on dendritic cells and tissue macrophages **(**Supplementary Fig. 8**)**.

**Figure 7:**
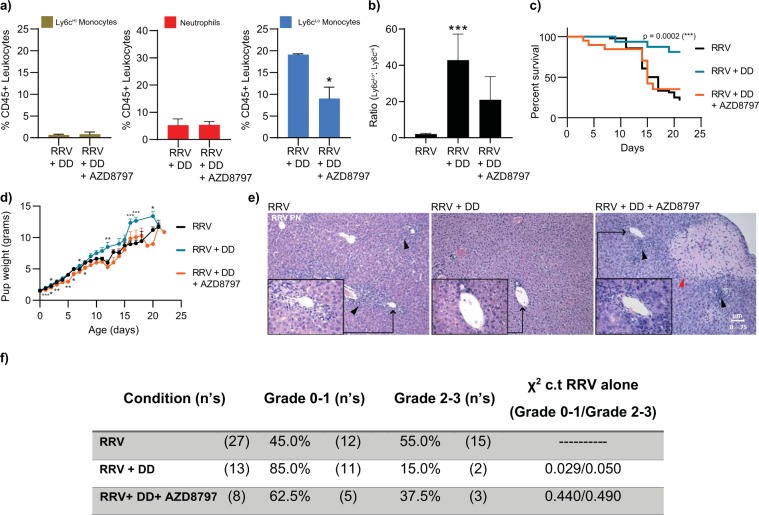
Inhibition of Ly6c^Lo^ non-classical monocytes restores susceptibility to RRV-mediated periportal inflammation. (**a–d**) Addition of AZD8797 had no effect on depleted Ly6c^Hi^ classical monocytes and neutrophils (n = 4), but did significantly reduce the Ly6c^Lo^ non-classical monocytes expansion observed in the DD group (p-value: 0.0303 (*)) and led to restoration of RRV susceptibility and observed weight loss (RRV n = 49, RRV_DD_ n = 16, RRV_DD + AZD8797_ n = 21). (**e**) Hematoxylin and eosin (H&E) stained sections demonstrate histological changes within the liver tissue of postnatal pups 3 weeks after injection with, (left to right): RRV only (n = 8), DD (n = 13), and DD + AZD8797 (n = 4). DD samples showed resolution of inflammation and absence of necrotic injury when compared to DD + AZD8797, which showed higher grade of inflammation and the presence of necrotic injury. Black arrows point to periportal inflammation. Red arrows point to necrotic foci. ((p-value: <0.05 (*), <0.01 (**), <0.001 (***)). Data represent mean +/−SEM. (**f**) Grading of periportal inflammation in liver tissue samples after RRV-mediated liver injury (n = 27), RRV + DD (n = 13), RRV + DD + AZD8797 (n = 5). Liver samples of pups injected with RRV + DD have less inflammation when compared to liver samples of pups injected with RRV alone. X^2^ c.t RRV alone = 0.029/0.050.

We then tested our hypothesis by depleting Ly6c^Hi^ monocytes and neutrophils in RRV-infected animals to see if the observed expansion of Ly6c^Lo^ monocytes conferred protection against RRV-mediated perinatal inflammation. Indeed, when both Ly6c^Hi^ classical monocytes and neutrophils were depleted, neonatal pups were rescued from RRV-mediated injury (Fig. 6j). Correspondingly, pup weights were significantly higher compared to RRV infection alone at time points when neonatal mice normally exhibit growth restriction due to RRV infection **(**Fig. 7d**)**. Furthermore, treatment with both anti-Ccr2 and anti-Ly6g led to a significant increase in the number of animals with grade 0–1 periportal inflammation and fewer mice with grade 2–3 inflammation compared to those treated with RRV alone, and in the majority of cases, there was near resolution of disease **(**Fig. 7e,f**)**. Survival rate after *either* Ly6c^Hi^ monocyte or neutrophil depletion was lower than for Ly6c^Hi^ monocyte and neutrophil-replete controls (Ly6c^Hi^ monocyte depletion: median survival: 14 days for RRV + Ly6c^Hi^ monocyte depletion vs. 17 days for RRV alone, p-value 0.0012; neutrophil depletion: median survival: 13 days for RRV + neutrophil depletion vs. 17 days for RRV alone, p < 0.0001, Fig. 6j), indicating that the absence of *both* neutrophils and Ly6c^Hi^ classical monocytes is necessary to confer resistance.

### Ly6c^Lo^ non-classical monocytes promote resolution of RRV-mediated liver inflammation

Although our results support the idea that expansion of Ly6c^Lo^ non-classical monocytes, in the experimental setting of Ly6c^Hi^ classical monocyte and neutrophil depletion, promotes resolution of neonatal periportal inflammation, we questioned whether the improvement in survival we observed was due to unopposed pro-reparative Ly6c^Lo^ monocyte function or whether it was due to the absence of pro-inflammatory Ly6c^Hi^ classical monocytes and neutrophils. Our transcriptional analysis of perinatal Ly6c^Lo^ non-classical monocytes supported the former, and we therefore hypothesized that inhibiting Ly6c^Lo^ monocytes in the setting of Ly6c^Hi^ monocyte- and neutrophil-depletion, would restore susceptibility to RRV infection. Based on the high level of Cx3cr1 expression in Ly6c^Lo^ non-classical monocytes^8,9^ we used the high-affinity Cx3cr1 small molecule inhibitor, AZD8797^23–25^, to test whether Cx3cr1 inhibition would reduce numbers of Ly6c^Lo^ non-classical monocytes and restore susceptibility to RRV-mediated inflammation **(**Fig. 6a**)**. Indeed, Ly6c^Lo^ non-classical monocyte proportions significantly decreased when AZD8797 was given in conjunction with anti-Ly6g and anti-Ccr2 **(**Fig. 7a**)**, resulting in a decrease in Ly6c^Lo^:Ly6c^Hi^ monocyte ratios **(**Fig. 7b**)**. These results are in agreement with the known mechanism of AZD8797 as a non-competitive modulator of Cx3cr1 that also inhibits downstream Cx3cr1-mediated G-protein signaling, thus resulting in a combination of diminished recruitment and function^25^. Other than its effects on Ly6c^Lo^ non-classical monocytes, AZD8797 caused a relative decrease in tissue macrophages, although this reduction did not result in a statistically significant difference **(**Supplementary Fig. 8**)**. Notably, when Ly6c^Lo^ non-classical monocytes were decreased in the absence of neutrophils and Ly6c^Hi^ monocytes, susceptibility to RRV-mediated inflammation was restored **(**Fig. 7c**)**. Survival after AZD8797 treatment was similar to survival after RRV alone and significantly lower than survival after Ly6c^Hi^ classical monocytes and neutrophil depletion (RRV + DD + AZD8797: median survival 15 days, p-value 0.80 c.t. RRV alone, p-value 0.008 c.t. RRV + DD**)**. Whereas RRV + DD pup weights were significantly higher compared to RRV infection alone at a time when neonatal mice exhibit growth restriction due to RRV infection, RRV + DD + AZD8797 pup weights decreased, similar to that observed in RRV alone **(**Fig. 7d**)**. Additionally, the grade of inflammation in the setting of DD + AZD8797 was similar to that seen after RRV alone **(**Fig. 7e,f**)**. These findings demonstrate that the abundance of Ly6c^Lo^ non-classical monocytes is associated with resolution of RRV-mediated inflammation, and that the reduction of these cells when AZD8797 is administered restores suseptibility to RRV-mediated injury. Although our data does not definitively prove that the disease resolution we observe with AZD8797 treatment occurs from its direct effect on Ly6c^Lo^ non-classical monocytes, these data support the idea that the abundance of Ly6c^Lo^ non-classical monocytes promotes resolution of RRV-mediated perinatal liver inflammation.

## Discussion

In this study, we hypothesized that inherent differences in Ly6c^Hi^ classical monocyte and Ly6c^Lo^ non-classical monocyte function, and their relative abundance in late gestation fetuses and neonatal pups, determine susceptibility to perinatal hepatic inflammation. Our results demonstrate that (i) Ly6c^Lo^ non-classical monocytes express a pro-reparative transcriptomic signature during the perinatal period and their abundance inversely correlates with susceptibility to RRV-mediated perinatal liver injury, (ii) the adaptive immune system and type 2 immunity play a limited role in inflammation after RRV infection, and (iii) experimental manipulation of Ly6c^Lo^ non-classical monocytes can render neonatal pups resistant to perinatal liver injury. Collectively, these results support the conclusion that Ly6c^Lo^ non-classical monocytes play an important role in promoting the resolution of RRV-mediated perinatal inflammation.

Individual immune subsets have been implicated as being important for the pathogenesis of RRV-mediated perinatal liver inflammation, including effector T cells^14,21,26,27^, regulatory T cells^28,29^, B cells^20^, natural killer cells^30^, and dendritic cells^31^. Using RAG KO mice, we examined the contribution of the adaptive immune system in the pathogenesis of RRV-mediated inflammation. The equivalent survival between RAG KO and wildtype animals during the first 14 days of injury suggests that the main contribution of the adaptive immune system to the resolution of inflammation is limited to later in the disease course. The similar degree of inflammation and survival we observed in type 2 immune deficient animals compared to wildtype animals does not eliminate the role of type 2 immunity during perinatal liver inflammation, as liver-specific cues or other type 2-activating pathways including IL-4 and the tissue alarmins Hmgb1, S100 family proteins, and IL-1alpha could play roles in disease pathogenesis.

A prime example of a disease involving neonatal liver inflammation is biliary atresia (BA), for which treatment options are limited due to our lack of understanding of the pathophysiology of perinatal liver inflammation in this setting. Although an immune-mediated component is thought to potentiate bile duct obstruction in patients with BA, non-specific immunosuppression through corticosteroids and antibody depletion through intravenous immunoglobulin have failed to demonstrate a difference in survival in patients with BA who undergo Kasai portoenterostomy^32–34^. Furthermore, these clinical interventions have been restricted to the postnatal environment and little is known about the prenatal factors that may contribute to susceptibility to disease, despite the compelling evidence that BA has fetal origins^35^. Our results demonstrate that the naturally occurring differences in inflammatory and reparative monocyte subsets during gestation may contribute to susceptibility and resilience to perinatal liver injury. The lack of clinical efficacy of corticosteroids in human BA could be further explained by the fact that glucocorticoid administration selectively inhibits human CD14^+^CD16^+^ non-classical monocytes^36^. We speculate that the underlying mechanisms that predispose patients to perinatal liver diseases like BA may be due to alterations in normal immune mechanisms that facilitate restoration of tissue homeostasis. Further work to define monocyte subsets and function in human perinatal liver inflammation will be needed to understand the role of Ly6c^Hi^ classical monocytes and Ly6c^Lo^ non-classical monocytes in human BA.

In addition to demonstrating a pro-reparative role for Ly6c^Lo^ non-classical monocytes during perinatal liver injury, our results also demonstrate that Ly6c^Lo^ non-classical monocytes expand and play a role in tissue repair even when Ly6c^Hi^ classical monocytes are depleted. Other models of tissue inflammation have demonstrated that Ly6c^Hi^ classical monocytes transition into Ly6c^Lo^ non-classical monocytes at the site of injury to support the restoration and repair of the tissue^37–39^. Our results challenge the concept that Ly6c^Hi^ monocytes serve as precursors to Ly6c^Lo^ monocytes;^38,40–42^ they suggest either that Ly6c^Lo^ non-classical monocytes develop without passing through the Ly6c^Hi^ classical monocyte state^43^ or alternatively, that only a fraction of Ly6c^Hi^ monocytes are required to maintain Ly6c^Lo^ monocytes^40^. Further work will be needed to determine whether the developmental trajectories of monocytes change in the setting of perinatal liver inflammation, and whether the fetal environment is conducive to a pro-reparative state through the preferential expansion of Ly6c^Lo^ non-classical monocytes.

One of the main limitations of the RRV model of periportal injury is that it is restricted to the Balb/c strain; RRV infection in C57BL/6 (B6) and 129 pups yields inconsistent and milder levels of inflammation (data not shown). As a result of its restricted phenotype in Balb/c animals and the predominance of transgenic immune deficient strains on the B6 background, manipulating immune populations is only possible after backcrossing transgenic strains on the B6 background to Balb/c, which can be a time-consuming endeavor. Alternative monocyte depletion strategies, including the use of clodronate liposomes, does not deplete monocytes in the liver despite efficient depletion of circulating monocytes (data not shown). Genetic strategies to render animals absent of Ly6c^Lo^ non-classical monocytes have been reported^44^, but we have found circulating Ly6c^Lo^ non-classical monocytes, albeit at lower frequencies, in Nr4a1-deficient animals (data not shown). Although use of AZD8797 did lead to the observation of a reduction of Ly6c^Lo^ non-classical monocytes along with restoration of susceptibility to RRV-mediated inflammation, based on the known expression of Cx3cr1 on monocyte-derived pro-inflammatory macrophages^45^, we cannot exclude the possibility that monocyte-derived macrophages play a role in RRV susceptibility. Our data, however, consistently demonstrates a survival benefit when Ly6c^Lo^ non-classical monocytes outnumber Ly6c^Hi^ classical monocytes in the setting of perinatal inflammation.

Here, we demonstrate that Ly6c^Lo^ non-classical monocytes promote resolution of RRV-mediated perinatal liver inflammation in both the physiologic environment of the late gestation fetus, and in the neonatal pup. These data support the use of targeted therapies that can enhance Ly6c^Lo^ non-classical monocyte function to mitigate the detrimental effects of perinatal liver inflammation.

## Methods

### Mice

Balb/c and C57BL/6 wildtype mice were obtained from the National Cancer Institute (Wilmington, MA). BALB/c.RAG KO (RAG KO) mice were purchased from Taconic Biosciences (Rensselaer, NY). All mouse experiments were approved by the UCSF Institutional Animal Care and Use Committee, and animals received humane care in accordance with the criteria outlined in the *Guide for the Care and Use of Laboratory Animals*.

### Creation of single-cell suspensions from fetal and neonatal livers

Livers and spleens were isolated from euthanized mice, washed with PBS and processed. To create single cell suspensions, spleens from all pups, regardless of age, and livers from pups younger than P7 were homogenized by gentle mechanical dissociation. Livers from pups older than P7 were dissociated using enzymatic digestion with 2.5 mg/ml Liberase (Roche, Indianapolis, IN, 05401119001) in 1 M CaCl_2_ HEPES Buffer.

### Single-cell RNA sequencing

For single-cell RNA sequencing, single cell suspensions were made from C57/BL6 mouse fetal livers at E15.5, E17.5, and P0. Single cells were sequenced with 10x Genomics’ droplet-based method^46^. CellRanger 2.1.1 was used to align raw reads and filter cell barcodes. Replicate samples were pooled together. Quality control, principal component analysis, clustering, and differential expression analysis were performed in R with Seurat (v3.0)^46^. Cells with less than 200 genes, unique molecular identifier count of less than 500 or greater than 20000, and greater than 25% of total expression from mitochondrial genes were excluded from analysis. The computational tool, SingleR (v0.2.2), was used to identify cell types in an unbiased fashion using reference data sets of transcriptomes derived from pure populations^10^. Based on this annotation, monocytes were identified and further sub-clustered. Monocytes with *Ly6c2* expression >1.5 (log-normalized counts) were classified as Ly6c^Hi^ classical monocytes; those with *Ly6c2* expression less than 1.5 (log-normalized counts) were classified as Ly6c^Lo^ non-classical monocytes. Differentially expressed genes between Ly6c^Hi^ classical monocytes and Ly6c^Lo^ non-classical monocytes, determined by a ≥ 0.5 log fold change and p < 0.05, were identified using the Wilcoxon Rank Sum test, and were functionally annotated using Reactome^11^.

### Flow cytometry

For flow cytometric analysis, liver and spleen single-cell suspensions were stained using the following antibodies: Cd11c, clone N418 (Biolegend, San Diego, CA, 117339); Ly6c, clone HK1.4 (Biolegend, 128035); MHCII, clone M5/114.15.2 (eBioscience, Waltham, MA, 48-5321-82); Cd45, clone 30-F11 (eBioscience, 56-0451-82); Cd11b, clone M1/70 (eBioscience, 47-0112-82); Cd64, clone X54-5/7.1 (BD Biosciences, Franklin Lakes, NJ, 741024); Ly6g, clone 1A8 (BD Biosciences, 560601); Fc block Cd16/Cd32, clone 2.4G2 (BD Biosciences, 553142); Ly6g, RB6-8c5 (Tonbo, San Diego, CA, 60-5931); Ghost (Tonbo, 13-0870-T100); Flow cytometric data was acquired on the BD LSRII Fortessa X20 and analyzed using FlowJo (Franklin Lakes, NJ).

### Postnatal and fetal models of perinatal inflammation

Rhesus rotavirus (RRV) and *Cercopithecus aethiops* kidney epithelial cells (MA104) cells were obtained from Dr. Henry Greenberg (Stanford University, Palo Alto, CA). Virus was grown and titered in MA104 cells. To induce postnatal hepatic inflammation, P0 pups were injected intraperitoneally with 1.5 ×10^6^ focus forming units (ffu) 24 hours after delivery. Postnatal animals were also treated with depleting antibodies for Ly6c^Hi^ classical monocytes (anti-Ccr2, clone MC21) at a dose of 2 mg/kg and neutrophils (anti-Ly6g, clone 1A8, BioXCell, West Lebanon, NH) at a dose of 5 mg/kg, or a small molecule inhibitor to Cx3cr1 to ablate Ly6c^Lo^ non-classical monocytes function (AZD8797, Axon Medchem, Reston, VA) at a dose of 16 mg/kg. For fetal injections, dams underwent survival surgery to expose the uterus and fetuses as previously described^16,17^. Fetuses received intrahepatic injections of 1.5 ×10^6^ ffu of RRV. Controls were injected with PBS using the same technique. Pups were weighed daily, and were observed for survival, activity, size, fur distribution, yellow discoloration of mucosal surfaces, and urine or stool color changes. Animals that appeared moribund were sacrificed.

### Viral titration assay

Samples of infected whole livers were weighed and homogenized using a glass tissue homogenizer in serum-free media. Ten-fold serial dilutions of liver homogenates were added to a confluent layer of MA104 cells grown in 6-well plates (1 sample/well). RRV stock and uninfected liver samples were used as positive and negative controls, respectively. Samples were incubated for 1 hour in 5% CO_2_ and 37 °C. After incubation, the liver homogenate was aspirated, and agarose in serum-free media (0.6% agarose, 2X M199, 0.45% NaHCO_3_, 0.5 ug/ml trypsin, 1% penicillin/streptomycin, 1% L-glutamine) were added to each well. Once agarose solidified, the plates were incubated in 5% CO_2_ and 37 °C. On the following 3^rd^ morning, plates were stained with 0.5% neutral red solution, incubated in 5% CO_2_ and 37 °C for 5 hours, and plaques were then counted. Viral titer per mg of tissue was calculated using the following equation: (# of plaques x viral volume x dilution factor)/sample weight.

### Histologic assessment of RRV-infected tissues

Hematoxylin and eosin tissue sections were assessed by a blinded board certified pathologist with specialization in Liver pathology. Histologic liver sections were assessed for presence of inflammation and fibrosis using the Batts-Ludwig methodology^47^. In addition, centrizonal necrosis and extramedullary hematopoiesis within the liver were assessed and graded using a range of 0 to 4.

### Statistics and data analysis

Graphpad Prism 8.0 (San Diego, CA) was used to generate graphs and perform statistical analysis. An unpaired t-test with Welch’s correction was used for comparing 2 groups. When comparing 2 groups that were not normally distributed, the non-parametric Mann-Whitney test was used. A one-way ANOVA with Tukey’s multiple comparisons test was used to compare multiple groups. The Chi-square test was used to compare differences in proportions between two groups. Survival was evaluated using the log-rank (Mantel-Cox) test. Quantification of myeloid populations after Ab-mediated depletion was calculated by dividing the number of live cells of interest by organ weight.

## Supplementary information

Supplementary information.

## Data Availability

The authors declare that all other data supporting the findings of this study are available within the article and its Supplementary Information Files.
