# Supplementary material for: Ly6cLo non-classical monocytes promote resolution of rhesus rotavirus-mediated perinatal hepatic inflammation
Source: Sci Rep. 2020 Apr 28;10:7165. doi: 10.1038/s41598-020-64158-2 (PMC7188847; doi:10.1038/s41598-020-64158-2)
Supplement: Supplementary file 1 — Supplementary information. [file 41598_2020_64158_MOESM1_ESM.pdf]

# Ly6c$^{Lo}$ non-classical monocytes promote resolution of rhesus rotavirus-mediated perinatal hepatic inflammation

Anas Alkhani MBBS,[1,4] Claire S. Levy PhD,[1,4] Margaret Tsui BS,[2,4] Katherine Rosenberg BA,[1,4] Katya Polovina BS,[1,4] Aras N. Mattis MD PhD,[3,4] Matthias Mack MD,[5] Steven Van Dyken PhD,[6] Bruce M. Wang MD,[2,4] Jacquelyn J. Maher MD,[2,4] and Amar Nijagal MD[1,4*]

**Contact information:** Anas Alkhani, anas.alkhani@ucsf.edu; Claire Levy, claire.levy@ucsf.edu; Margaret Tsui, margaret.tsui@ucsf.edu; Katherine Rosenberg, katherine.rosenberg@ucsf.edu; Katya Polovina, katya.l.polovina@gmail.com; Aras N. Mattis, aras.mattis@ucsf.edu; Matthias Mack, matthias.mack@klinik.uni-regensburg.de; Steven Van Dyken, svandyken@wustl.edu; Bruce M. Wang, bruce.wang@ucsf.edu; Jacquelyn J. Maher, jacquelyn.maher@ucsf.edu; Amar Nijagal, amar.nijagal@ucsf.edu

**Affiliations**: Departments of Surgery,[1] Medicine,[2] Pathology,[3] and the Liver Center,[4] University of California, San Francisco, CA; Regensburg University Medical Center, Regensburg, Germany[5]; Department of Pathology and Immunology, Washington University School of Medicine, Saint Louis, MO[6].

[*]**Corresponding Author:**

Amar Nijagal, MD

Assistant Professor of Surgery

Division of Pediatric Surgery

513 Parnassus Avenue, HSW 1652, Campus Box 0570

University of CA, San Francisco

San Francisco, CA 94143-0570

Office: 415-476-4086; Fax: 415-476-2314

Email: Amar.Nijagal@ucsf.edu

**SUPPORTING INFORMATION**

**METHODS**

*Mice*

$Crlf2^{-/-}Il25^{-/-}Il1r1^{-/-}$(TKO) and wild type ($WT^{A}$) carrying $Arg1^{Yarg}$ and $Il4^{4get}$ reporter alleles on the Balb/c background were generated as previously described [1-3]. All mouse experiments were approved by the UCSF Institutional Animal Care and Use Committee, and animals received humane care in accordance with the criteria outlined in the *Guide for the Care and Use of Laboratory Animals*.

**SUPPLEMENTARY FIGURE LEGENDS**

**Supplementary Fig. 1:** *Ly6c2 is predominantly expressed in fetal and neonatal monocytes and granulocytes.* Violin plots demonstrate the distribution and differential gene expression of *Ly6c2* in immune, stromal, and parenchymal populations in the fetal and neonatal liver at E15.5, E17.5, and P0.

**Supplementary Fig. 2**: *Ly6c$^{Hi}$ classical and Ly6c$^{Lo}$ non-classical monocytes demonstrate differential expression of Ly6c2, Cx3cr1, Cd62l, and Ccr2*. Box and whisker plots show the expression of *Ly6c2, Cx3cr1, Cd62l (Sell),* and *Ccr2* among Ly6c$^{Hi}$ and Ly6c$^{Lo}$ monocytes at the following fetal and perinatal time points: (a) E15.5, (b) E17.5, and (c) P0. *Cx3cr1* expression was higher in Ly6c$^{Lo}$ non-classical monocytes, specifically in the late-gestation fetus. Ly6c$^{Hi}$ classical monocytes exhibited higher expression of both *Cd62l (Sell)* and *Ccr2*. The bottom boundary of the box represents the 25th percentile, the line within the box represents the median, and the upper boundary of the box represents the 75th percentile. Whiskers above and below the boxes indicate the maximum and minimum values, respectively, up to 1.5 times the interquartile range. Any data points out of this range are plotted as outliers. Statistical comparison was completed using the Mann-Whitney test. (p-value: < 0.05 (*), <0.01 (**), <0.0001 (****)).

**Supplementary Fig. 3:** *Expression of pro-inflammatory and anti-inflammatory/pro-reparative genes in Ly6c$^{Hi}$ classical and Ly6c$^{Lo}$ non-classical monocytes.* Box and whisker plots show the expression of (a) anti-inflammatory/pro-reparative genes and (b) pro-inflammatory genes in Ly6c$^{Hi}$ classical monocytes and Ly6c$^{Lo}$ non-classical monocytes at E15.5, E17.5, and P0. The expression of *Tgfb1* and *Il4ra*, which are both implicated in the polarization of M2 anti-inflammatory macrophages, was elevated in Ly6c$^{Lo}$ non-classical monocytes compared to Ly6c$^{Hi}$ classical monocytes. In Ly6c$^{Hi}$ classical monocytes, there was higher expression of the pro-inflammatory genes *S100a1*, *Mmp8*, interferon-gamma receptors *Ifngr1* and *Ifngr2*, interferon-related genes *Irf5*, *Ifi204*, *Ifitm2*, *Ifitm3*, and *Ifitm6*, and genes involved with NOD-like receptors, *Naip2*, *Casp1*, and *Card9*. See Supplementary Figure 2 legend for explanation of box

plots. Mean gene expression for each group is indicated by a black point within each box. Statistical comparison was completed using the Mann-Whitney test. (p-value: < 0.05 (*), <0.01 (**), <0.001(***), <0.0001 (****)).

**Supplementary Fig. 4:** *Myeloid immune populations in the perinatal liver and spleen.* Percent $Cd45^+$ leukocytes and absolute cell count of (a) liver tissue macrophages (tissue macs), (b) liver dendritic cells (cDC), (c) liver neutrophils, (d) spleen tissue macs, (e) spleen cDC, and (f) spleen neutrophils. Age (n): E17.5 (n=15); P0 (n≥6); P3 (n=6); P7 (n=9); P10 (n=6); P14 (n≥4). Data represent mean + SEM.

**Supplementary Fig. 5**: *RRV infection results in liver-specific inflammation.* Hematoxylin & Eosin stains of a representative RRV-injected pup at P14 (left) and PBS-injected control at P14 (right) showing sections from the liver, spleen, kidney, brain, heart, lungs, small intestine, and large intestine (n=1 each for every organ). All tissues except the liver showed no significant histological changes in RRV-injected pups, indicating that RRV-mediated injury is liver-specific in Balb/c mice.

**Supplementary Fig. 6**: *Type 2 immunity is not required for establishing disease after RRV infection.* (a) RRV infection in the Tslpr, Il-25, and Il-33r deficient mouse (TKO, $Crlf2^{-/-}Il25^{-/-}Il1r1^{-/-}Arg1^{Yarg/Yarg}$) was compared to $Arg1^{Yarg/Yarg}$ wild type ($WT^A$) controls. Kaplan-Meier survival of RRV-infected TKO (n=32) and $WT^A$ (n=43) mice. Both TKO (p-value 0.49) and $WT^A$ (p-value 0.53) had equivalent survival after RRV infection. (b) Weight over time of TKO pups injected with RRV (n=32) was compared to $WT^A$ pups injected with RRV (n=43). There was no significant difference between the weights of both strains after RRV injection. Data represent the mean +/-SEM. (c) Hematoxylin and eosin (H&E) stained sections demonstrate histological changes within the liver tissue of postnatal pups two weeks after injection with RRV, (left to right): $WT^A$ (n=4) and TKO (n=6). Black arrows point to periportal inflammation in liver samples after RRV-mediated liver injury. Red arrow points to necrotic foci in liver samples after RRV-mediated liver injury.

**Supplementary Fig. 7**: *Effect of anti-Ccr2 and anti-Ly6g depletion on myeloid populations.* Representative flow plots, each population as a proportion of $Cd45^+$ leukocytes, and absolute cell count for pups infected with RRV and then treated with either a) anti-Ccr2 or its isotype, IgG2b, to characterize the depletion of $Ly6c^{Hi}$ classical monocytes, ($n_{IgG2b}$=2, $n_{anti\text{-}Ccr2}$=4, $neutrophils_{\%Cd45+}$ p-value: 0.0002 (***), $Ly6c^{Lo}$ non-classical $monocytes_{\%Cd45+}$ p-value: 0.0283 (*), $cDCs_{\%Cd45+}$ p-value: <0.0001 (****), $neutrophils_{Absolute\ Cell\ Count}$ p-value: 0.0167 (*), $Ly6c^{Hi}$ classical $monocytes_{Absolute\ Cell\ Count}$ p-value: <0.0001 (****)) and b) anti-Ly6g or its isotype, IgG2a, to characterize the depletion of neutrophils ($n_{IgG2a}$=4, $n_{anti\text{-}Ly6g}$=4, $neutrophils_{\%Cd45+}$ p-value: 0.0193 (*), $Ly6c^{Lo}$ non-classical $monocytes_{Absolute\ Cell\ Count}$ p-value: 0.0260 (*)). Data represent mean $\pm$ SEM. (p-value: < 0.05 (*), <0.001(***), <0.0001 (****)).

**Supplementary Fig. 8**: *The administration of anti-Ly6g, anti-Ccr2, and the Cx3cr1 small molecule inhibor, AZD8797, does not cause significant changes to tissue macs or cDCs during RRV infection.* The percentage of tissue macrophages and dendritic cells in the liver ($n_{RRV}$=18) and spleen ($n_{RRV}$=14) after DD (n=4) and DD+ AZD8797 (n=4). Data represent mean $\pm$ SEM.

**REFERENCES**


1. Van Dyken, S. J. *et al.* Chitin activates parallel immune modules that direct distinct inflammatory responses via innate lymphoid type 2 and γδ T cells. *Immunity* **40**, 414–424 (2014).

2. Reese, T. A. *et al.* Chitin induces accumulation in tissue of innate immune cells associated with allergy. *Nature* **447**, 92–96 (2007).

3. Van Dyken, S. J. *et al.* A tissue checkpoint regulates type 2 immunity. *Nat. Immunol.* **17**, 1381–1387 (2016).

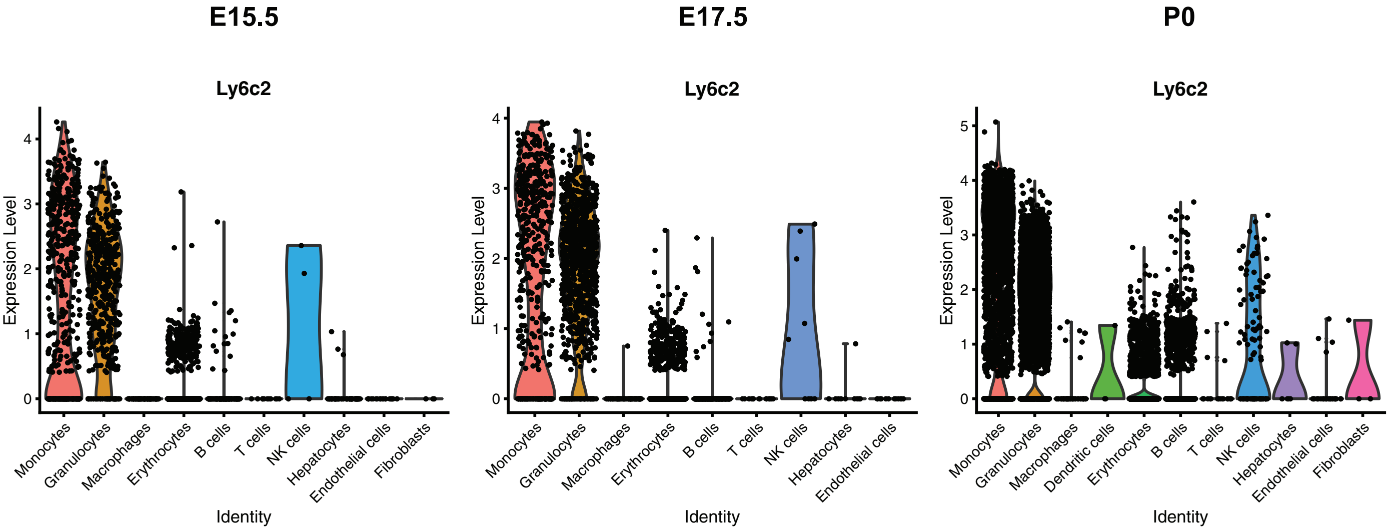


**Supplementary Figure 1**

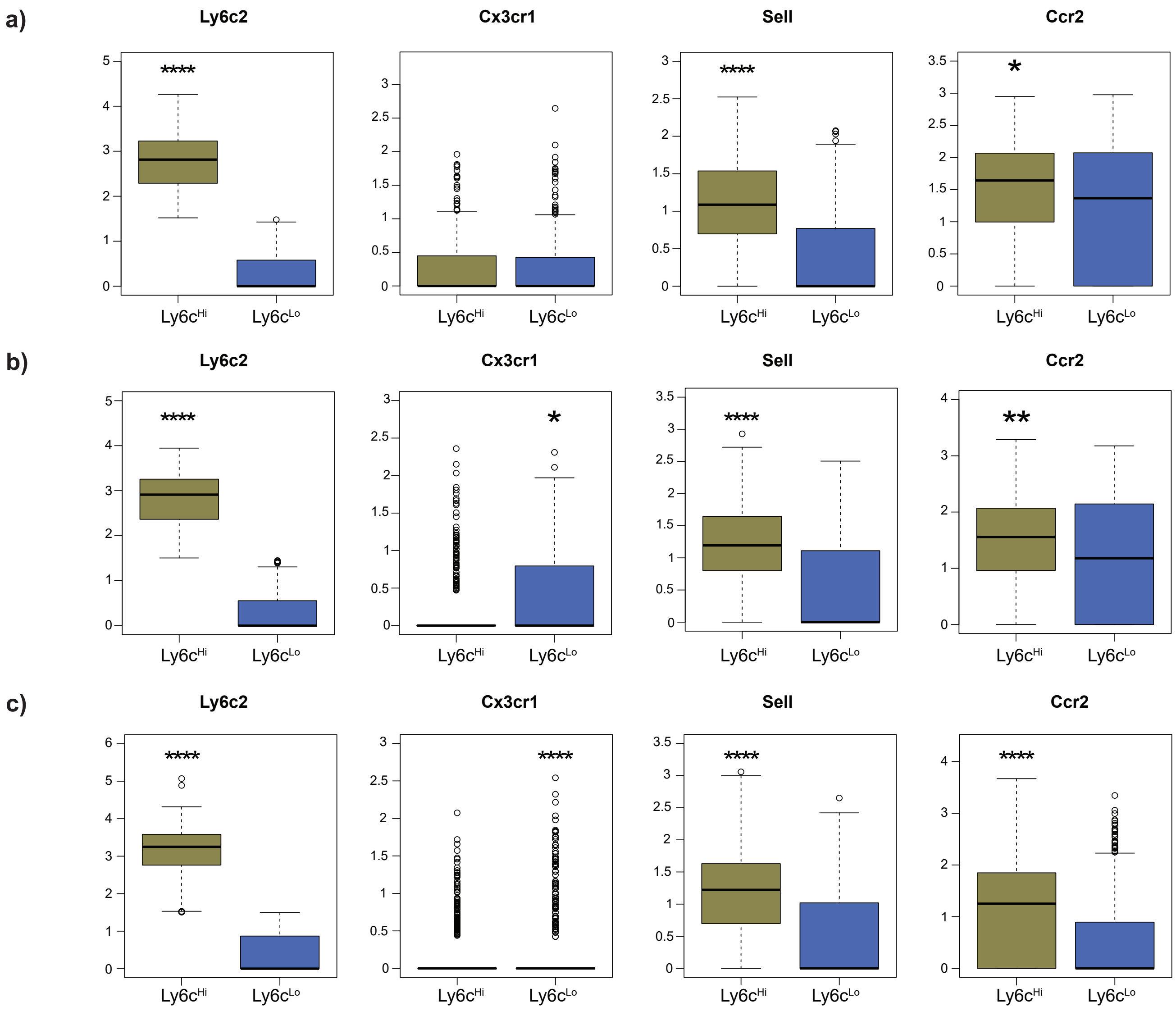


**Supplementary Figure 2**

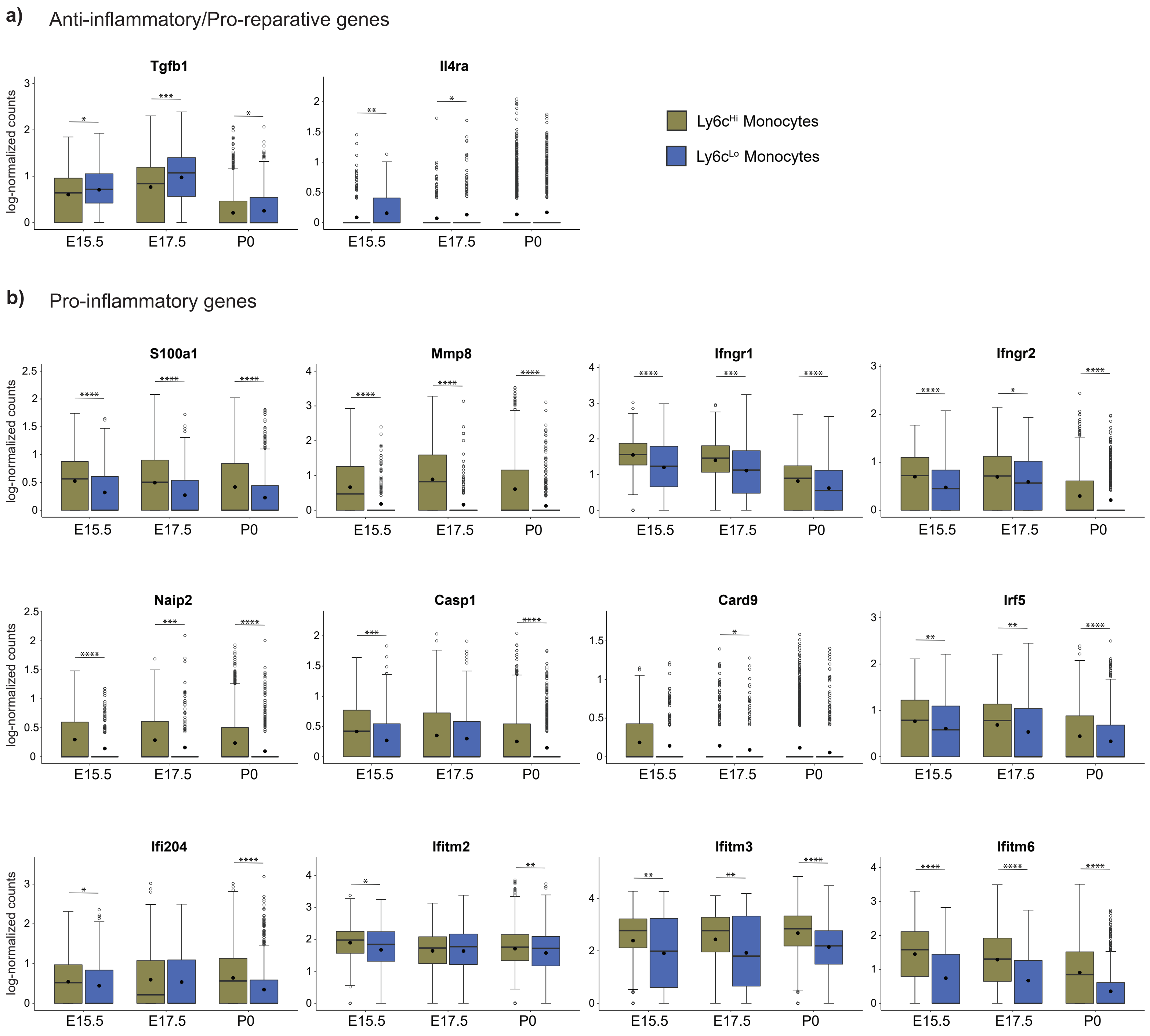


**Supplementary Figure 3**

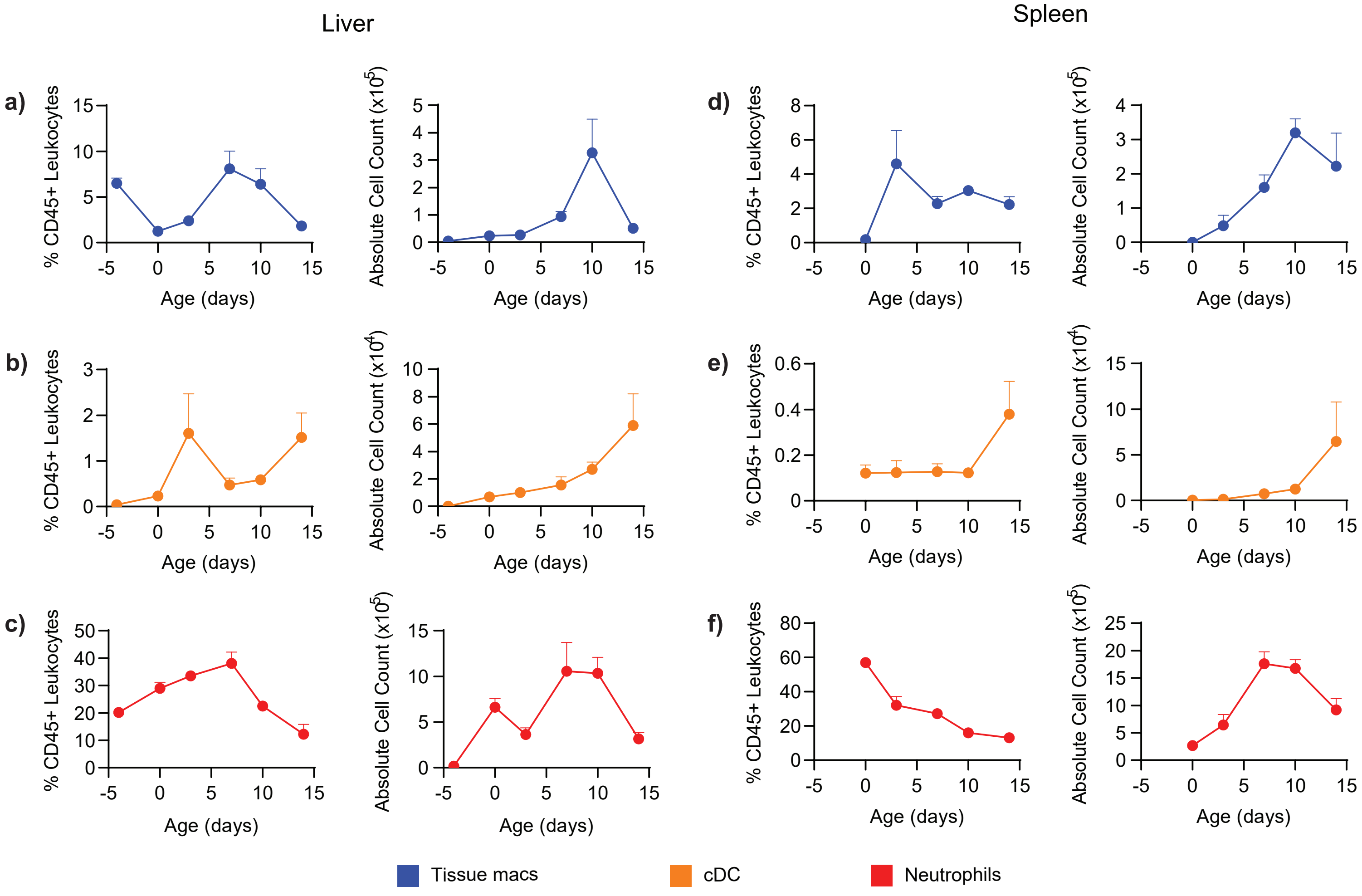


**Supplementary Figure 4**

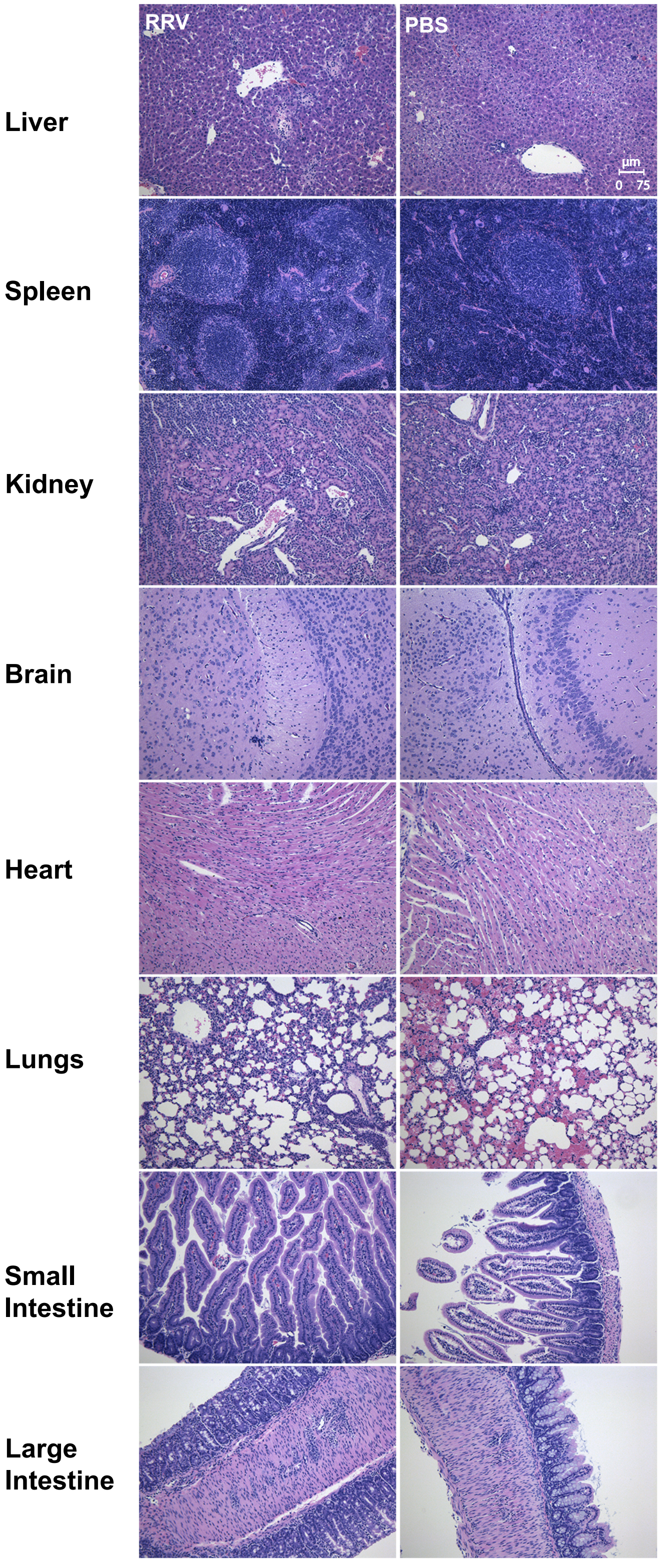


Supplementary Figure 5

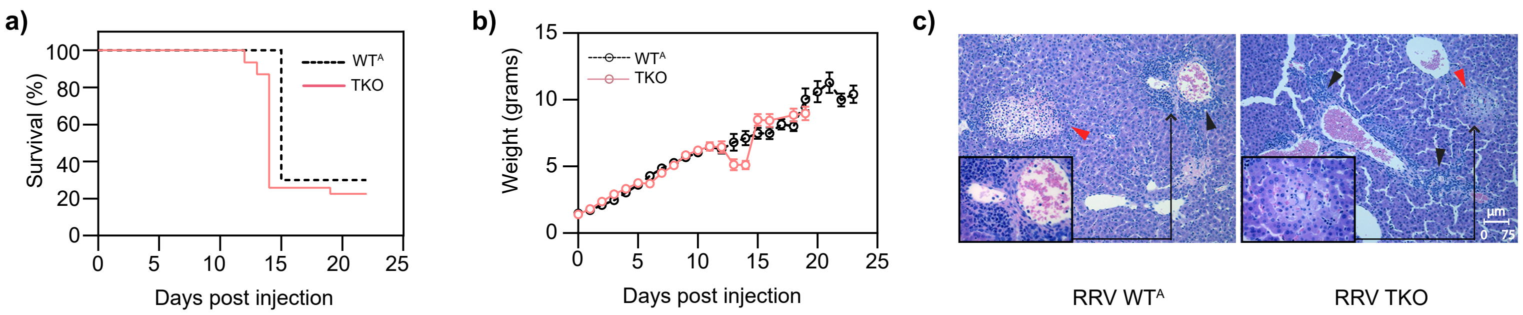


**Supplementary Figure 6**

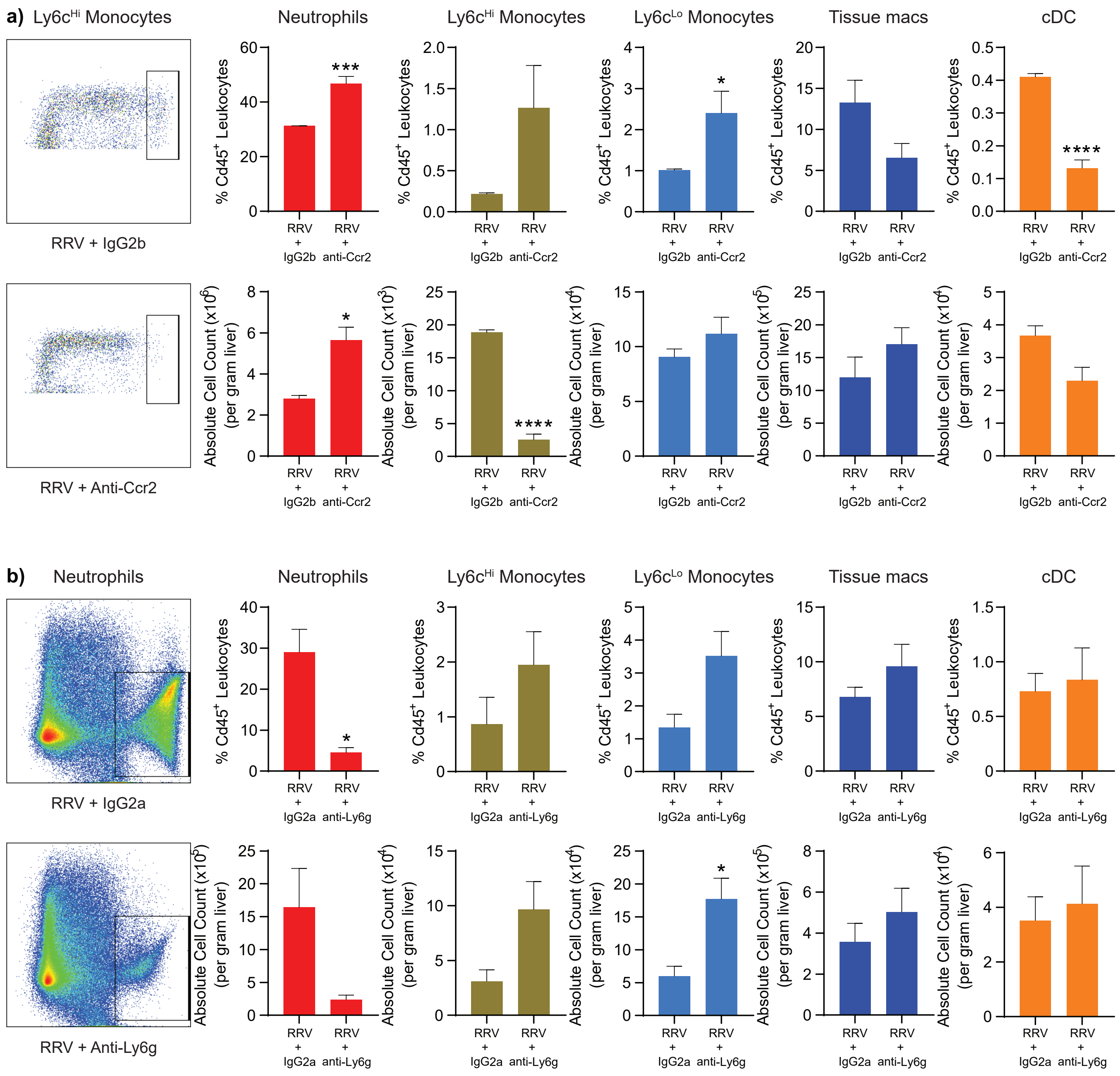


**Supplementary Figure 7**

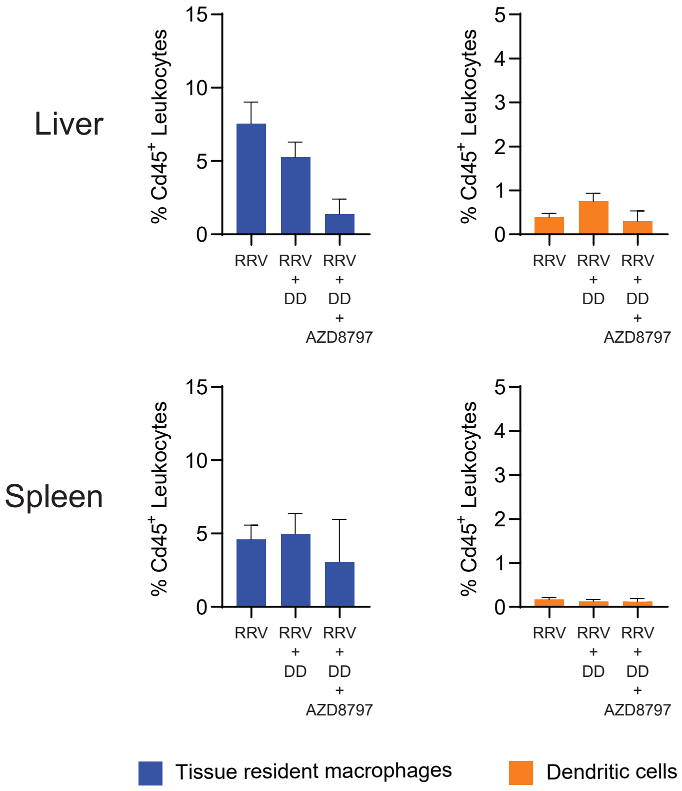


**Supplementary Figure 8**
